# Association between 19-bp Insertion/Deletion Polymorphism of Dopamine β-Hydroxylase and Autism Spectrum Disorder in Thai Patients

**DOI:** 10.3390/medicina58091228

**Published:** 2022-09-06

**Authors:** Wikrom Wongpaiboonwattana, Areerat Hnoonual, Pornprot Limprasert

**Affiliations:** 1 Faculty of Medicine, King Mongkut’s Institute of Technology Ladkrabang, Bangkok 10520, Thailand; 2 Department of Pathology and Genomic Medicine Center, Faculty of Medicine, Prince of Songkla University, Songkhla 90110, Thailand

**Keywords:** autism spectrum disorder, 19-bp ins/del, dopamine beta-hydroxylase

## Abstract

*Background and Objectives*: Autism spectrum disorder (ASD) is a neurodevelopmental disorder the cause of which is not fully known. Genetic factors are believed to play a major role in the etiology of ASD. However, genetic factors have been identified in only some cases, and other causes remain to be identified. This study aimed to identify potential associations between ASD and the 19-bp insertion/deletion polymorphism in the dopamine beta-hydroxylase (*DBH*) gene which plays a crucial role in the metabolism of neurotransmitters. *Materials and Methods*: The 19-bp insertion/deletion polymorphism upstream of the *DBH* gene was analyzed for associations in 177 ASD patients and 250 healthy controls. Family-based analysis was performed in family trios of each patient using the transmission disequilibrium test to investigate the potential contributions of this *DBH* polymorphism to ASD. *Results*: The frequency of the 19-bp insertion allele was significantly higher in the patient group compared to the controls (0.624 vs. 0.556, respectively; *p* = 0.046). The frequency of the insertion/insertion genotype was also higher in the patient group (0.378 vs. 0.288, respectively) but without statistical significance (*p* = 0.110). The family-based analysis showed an association between patient families and the insertion allele when only families of male participants were analyzed (73 vs. 48 events; OR 1.521; 95% CI 1.057–2.189; *p* = 0.023). *Conclusions*: This population-based analysis found an association between the 19-bp insertion allele of the *DBH* gene and ASD. No association at the genotype level was found. The family-based analysis found an association between the insertion allele and ASD when the analysis was performed on male participants only, suggesting a linkage between the *DBH* locus and ASD.

## 1. Introduction

Autism spectrum disorder (ASD) is a neurodevelopmental disorder manifesting primarily in children with limited social interactions and communication and restricted, repetitive behaviors as core features [1]. The cause of ASD is yet to be determined but is believed to be multifactorial with strong genetic influences [2]. The global prevalence of ASD is around 1% with a 3–4 times higher risk in males than females [3,4]. Studies identifying the genetic causes of ASD in various populations are required to build up a more complete picture of the disease.

**Figure 1:**
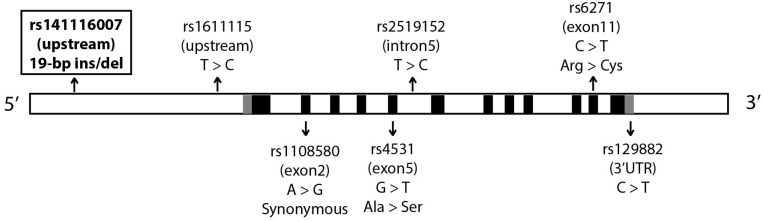
Variants on *DBH* associated with ASD and other neurological diseases. (Black boxes = coding sequences; gray boxes = untranslated regions (UTRs); the diagram is not to scale).

The dopamine beta-hydroxylase (*DBH*) gene translates a rate-limiting enzyme in dopamine metabolism that catalyzes the conversion of dopamine to norepinephrine. Dopamine, norepinephrine, and DBH enzyme have been suggested to be related to ASD and autistic-like behaviors [5,6,7,8]. The *DBH* gene is also related to other neurological diseases such as schizophrenia and Alzheimer’s disease [9,10]. Many variants of the *DBH* gene have been found to be associated with plasma dopamine levels and DBH activity (Figure 1) [11,12,13,14]. Several studies have identified associations between *DBH* polymorphisms and mothers of ASD patients or symptoms of ASD [15,16]. Many polymorphisms in the *DBH* gene such as rs1611115, rs2519152, and rs6271 have been shown to have functional effects on the DBH enzyme and might be a cause of ASD [14]. However, this study focused on the polymorphism of 19-bp insertions (ins) or deletions (del) (rs141116007) in the intergenic region upstream of *DBH.* There is evidence from various studies that this polymorphism is associated with DBH activity in serum and plasma [14,17,18]. In vitro expression by a reporter assay using recombinant plasmids with the del allele gave lower expression levels than the 19-bp ins allele [19]. However, the limited number of studies on the ASD population makes conclusions about this polymorphism elusive. Therefore, this study aimed to identify associations of the 19-bp ins/del polymorphism in Thai ASD patients.

## 2. Materials and Methods

### 2.1. Subjects

Based on the Diagnostic and Statistical Manual of Mental Disorders, Fourth Edition (DSM-IV) criteria, 177 ASD patients (149 males and 28 females) were recruited under an ASD genetic screening project. The ASD diagnoses were based on the DSM-IV criteria for autistic disorder and pervasive developmental disorders–not otherwise specified (PDD-NOS) [20] and comprised 136 with autistic disorder (114 males, 22 females) and 41 with PDD-NOS (35 males, 6 females). Their ages ranged from 17 months to 16 years and 7 months (average 51.8 months), with non-verbal IQ scores ranging from 41 to 127 (average 66.4, <70 = 62.8%) by using the Stanford–Binet Intelligence Scale: Fifth edition (Stanford-Binet-V). We excluded known syndromes and non-genetic risk factors of ASD (i.e., low birth weight, congenital malformations, perinatal asphyxia, and perinatal infection). Nine patients (5.2%) presented with suspected attention-deficit and hyperactivity disorder (ADHD). This study also excluded one patient with ring chromosome 13 [21], patients with pathogenic copy number variations, variants of uncertain significance, or likely pathogenic copy number variations identified by chromosomal microarray [22]. Patients with clinically significant variants identified by whole-exome sequencing were also excluded [23]. Karyotyping confirmed normal chromosomes in all 177 patients. Mutation screening for Fragile X syndrome and *MECP2* mutations was confirmed negative before the study. Out of the 177 patients, the parents of 151 patients were also recruited. The 250 normal controls (178 males and 72 females) were recruited from our previous study [24]. All participants signed written consent forms. Ethical approval for a human subject study was granted by the Institutional Review Board of the Faculty of Medicine, Prince of Songkla University (EC48/364-006 and EC48/364-006-3).

### 2.2. Identification of 19-bp Ins/Del Polymorphism

Blood samples were collected from all study participants, patients and controls, and analyzed for the 19-bp ins/del polymorphism with PCR adapted from a previous report [25]. The reaction contained 1 µM of the primers: 5′-GCAAAAATCAGGCACATGCACC-3′ and 5′-CAATAATTTGGCCTCAATCTTGG-3′, 1 mM MgCl_2_, 0.1 mM dNTPs, 1 U Invitrogen Taq DNA polymerase, and 50 ng of DNA template. The PCR condition began with 5 min of 95 °C initial denaturation followed by 30 cycles of 30 s of 95 °C denaturation, 30 s of 58 °C annealing, 1 min of 72 °C extension steps, and finally 7 min of 72 °C final extension. The PCR products were visualized with 8% acrylamide gel. The products of 163 bp and 144 bp were considered ins and del alleles, respectively.

### 2.3. Data Analysis

Hardy–Weinberg equilibrium was used to assess data quality. Association analysis and the transmission disequilibrium test were performed to test for associations. Statistical tests of allele and genotype frequencies between patients and controls and transmission disequilibrium in patient family trios were performed with the Chi-square test. Odds ratios were calculated by the odds of ins allele transmission over the odds of del allele transmission. All statistical tests were performed using PLINK 1.9 software (http://www.cog-genomics.org/plink2/) (Accessed on 30 May 2022) [26].

## 3. Results

The Hardy–Weinberg equilibrium was used to assess the controls, and a random distribution of alleles was confirmed (*p* = 0.201). The allele and genotype frequencies of the 19-bp ins/del polymorphism are shown in Table 1. A significant difference in allele frequencies between the patients and controls was observed (χ^2^ = 3.980, *p* = 0.046). A higher ins allele frequency and lower del allele frequency were observed in the patients compared to the controls (0.624 vs. 0.556 for the ins allele, 0.376 vs. 0.444 for the del allele). However, no significant difference was observed in the genotype frequencies (χ^2^ = 4.406, *p* = 0.110). A slightly higher frequency of the ins/ins genotype was observed in the patients over the controls (0.378 vs. 0.288), but lower frequencies of the ins/del and del/del genotypes were found in patients when compared with the control group (0.492 vs. 0.536 for the ins/del genotype, 0.130 vs. 0.176 for the del/del genotype). When male patients and controls were analyzed separately, a similar trend of the ins allele being more frequent in the patient group was observed (0.641 vs. 0.573). However, no significant differences in allele frequencies (χ^2^ = 3.127, *p* = 0.077) or genotype frequencies (χ^2^ = 3.706, *p* = 0.157) were found.

Family-based analysis of allele transmission from parents to the affected child using the transmission disequilibrium test showed that the ins allele occurred slightly more often than the del allele in families with an ASD child (84 vs. 64 events; OR 1.312; 95% CI 0.948–1.817; *p* = 0.100) but without statistical significance (Table 2). However, when only families of male participants were analyzed, there was a significant difference, indicating that the ins allele was transmitted more than the del allele in this group (73 vs. 48 events; OR 1.521; 95% CI 1.057–2.189; *p* = 0.023).

## 4. Discussion

An association between the 19-bp ins allele and ASD patients was found in both the population-based and family-based methods in this study. To our knowledge, this is the first study to find an association between the 19-bp ins/del polymorphism and ASD. A previous study in Canada found a relationship between this polymorphism and mothers of ASD patients but no association with ASD children [18]. Another study in the United States, however, found no association between this polymorphism and ASD [27]. Studies on migraine and schizophrenia found associations between the del allele and the diseases or symptoms in the patients [28,29,30,31]. However, meta-analyses examining studies conducted comparing this polymorphism and schizophrenia and migraine found no association [32,33]. 

Previous studies have suggested that the ins allele is associated with increased DBH activity [14,17,19]. However, ASD was found to be associated with decreased DBH activity [8]. It is possible that DBH activity is minimally affected by this polymorphism, as a previous study found other variants in the *DBH* gene (rs1611115, rs2519152, and rs6271) which could explain dopamine levels better than the 19-bp ins/del polymorphism [14]. The data obtained in our study might represent a population-specific characteristic of the Thai population in which only the 19-bp ins/del polymorphism itself might have a minimal effect on DBH activity. However, this polymorphism might form a linkage disequilibrium with other loci that contribute more to the DBH activity. A previous study identified linkages between multiple loci near the *DBH* gene and plasma DBH activity suggesting a multiple-site contribution rather than a single-variant contribution [34].

Genetic variations in the *DBH* gene are closely linked to many neurological diseases. Apart from the 19-bp ins/del polymorphism, other variants such as rs1611115 have been associated with plasma DBH activity in schizophrenia [9] and are a possible biomarker in cerebrospinal fluid for Alzheimer’s disease [10]. A study in Spain also found a contribution of rs1611115 to phonological awareness and syllable recognition, which are related to dyslexia or attention-deficit hyperactivity disorder (ADHD) [35]. An rs6271 polymorphism in the *DBH* gene has also been associated with schizophrenia in a case-control and family-based study from India [36] and bipolar disorder in a study in Turkey [37]. A synonymous variant in exon 2, rs1108580, has been associated with cognitive function in schizophrenia patients [9]. A significant correlation between rs1108580 and low DBH activity has also been observed in male ADHD patients [38]. A variant in a 3′ untranslated region, rs129882, has been associated with Parkinson’s disease in East India, and it also influenced the association in the C–A–T haplotype (rs1611115–rs1108580–rs129882) of the *DBH* gene [39]. These findings support that the genetic variants of the *DBH* gene are closely related to neurodevelopmental processes and could be potential contributing factors in ASD pathogenesis.

In the proximal region of the *DBH* gene, there are tuberous sclerosis 1 (*TSC1*) and retinoid × receptor α (*RXRA*) genes which are located approximately 700 kbp upstream and downstream, respectively. These genes could form a linkage with the 19-bp ins/del polymorphism. The TSC1 protein is a post-translational regulator of the mammalian target of the rapamycin (mTOR) signaling pathway which controls the growth and metabolism of the nervous and other systems. TSC1 forms a protein complex with the tuberous sclerosis 2 (TSC2) protein and acts as a suppressor of the mTOR signaling pathway [40]. Mice deficient in *TSC1* have increased mTOR activity and ASD-like behaviors [41]. Mutations in *TSC1* and *TSC2* are known to cause syndromic autism, known as tuberous sclerosis, with high rates of comorbid ASD. Novel coding *TSC1* variants have been previously reported in some ASD cases [42,43] supporting a link between *TSC1* and ASD. The *RXRA* gene encodes retinoid × receptor (RXR) α isotype which is part of the RXR family working as a nuclear receptor. It forms a homodimer or heterodimer with other nuclear receptors including retinoic acid receptor (RAR) [44]. Mutations in the coactivator complex of the *RAR/RXR* gene, known as arginine–glutamic acid dipeptide repeats (*RERE*), have been identified in patients with syndromic intellectual disability and autistic features [45]. RXR is a part of the retinoic acid pathway, the major pathway regulating neural differentiation, and has an important contribution to ASD [46]. In addition, lower levels of retinoic acid were found in Chinese patients with ASD [47]. Since these two genes are related to ASD and located near the *DBH* gene, they could be linked with the 19-bp ins allele in the Thai population and be the explanation for why the linkage disequilibrium was found in this study.

Recently, a hypothesis for explaining dopaminergic dysfunction in ASD was proposed, which pointed out possible connections between dopamine and the midbrain dopaminergic system [7]. Altered levels of dopamine can affect sensorimotor networks and salience networks through mesocorticolimbic and nigrostriatal pathways. Not only dopamine but other neurotransmitters as well, such as serotonin, can stimulate or inhibit networks within the midbrain dopaminergic system [48]. Our previous study also showed an association between ASD and serotonin receptor polymorphism in Thai patients suggesting a cumulative genetic effect from multiple loci might contribute to ASD [49].

The contribution of the 19-bp ins/del polymorphism might have a minimal effect on DBH activity in Thai patients but could also be linked to other regions which contribute more to the DBH activity. Other variants in the *DBH* gene such as rs1611115 or rs6271 could be additional factors related to DBH activity and ASD. The genes nearby the *DBH* gene could also be the factors causing ASD in Thai patients. Larger sample sizes with more than one variant in the *DBH* gene and nearby genes should be examined to further elucidate any links between the *DBH* gene and ASD.

Even though the data suggested an association between 19-bp ins/del polymorphisms and ASD, there are limitations to the interpretation of these results. The significant association only at the allele frequency but not the genotype frequency suggests that such associations could have occurred randomly during the study by some difficult-to-control factors such as sampling bias or population stratification. The transmission disequilibrium test was performed to overcome these factors. The data on the family-based analysis showed significant differences only when male patients were analyzed, which could be a result of the small sample size, and the insignificant results of the pooled analysis could be real. However, since ASD occurs more frequently in males than females with the cause not fully understood, a separate analysis between males and females would be better to reveal the relationship between the 19-bp ins/del polymorphisms and ASD. In addition, the associations found in this study could be comorbidities of ADHD and ASD since genetic relationships between ADHD and ASD have been reported [50]. A few variants in the *DBH* gene have also been reported to be associated with ADHD [51,52]. At least 5.2% of the patients in this study had come to the clinic with ADHD. This observation might raise the question of whether the association and significant transmission disequilibrium found could be a relationship between the *DBH* gene and ASD or a subgroup of patients with a comorbidity of ADHD. However, there were not enough clinical data on ADHD in our cohort study. In order to verify this possible association, studies with larger sample sizes together with comorbidity data and other candidate genes associated with ASD should be performed. 

## 5. Conclusions

Association was found between the 19-bp ins/del polymorphism of the *DBH* gene and ASD in the Thai population. However, the significance of the association was small, indicating the association could be simply by chance. The transmission disequilibrium test showed the association between the ins allele and ASD when only male participants were observed. The latter finding is very intriguing because it possibly shows a linkage between the *DBH* locus and ASD. Further studies with a larger sample size and other variants in the *DBH* gene should be performed to elucidate this finding.

## Figures and Tables

**Table 1:** Allele and genotype frequencies of the 19-bp ins/del alleles in the *DBH* gene compared between patients and normal controls.

Participants (No. of Patients/Controls)	Allele or Genotype	Patients		Controls		χ^2^	*p*-Value
		Number	Frequency	Number	Frequency		
All participants (177/250)	ins	221	0.624	278	0.556	3.980	0.046 *
	del	133	0.376	222	0.444		
	ins/ins	67	0.378	72	0.288	4.406	0.110
	ins/del	87	0.492	134	0.536		
	del/del	23	0.130	44	0.176		
Male participants (149/178)	ins	191	0.641	204	0.573	3.127	0.077
	del	107	0.359	152	0.427		
	ins/ins	59	0.396	53	0.298	3.706	0.157
	ins/del	73	0.490	98	0.550		
	del/del	17	0.114	27	0.152		

* *p* < 0.050.

**Table 2:** Transmission disequilibrium test results between ins and del alleles in patient families.

Participants (No. of Families)	Allele	T	NT	OR (95% CI)	χ^2^	*p*-Value
All participants (151)	ins	84	64	1.312 (0.948–1.817)	2.703	0.100
	del	64	84			
Male participants (128)	ins	73	48	1.521 (1.057–2.189)	5.165	0.023 *
	del	48	73			

* *p* < 0.050; ins: insertion allele; del: deletion allele; T: transmitted; NT: not transmitted; OR: odds ratio; CI: confidence interval.

## Data Availability

Not applicable.
